# Distance to clinic is a barrier to PrEP uptake and visit attendance in a community in rural Uganda

**DOI:** 10.1002/jia2.25276

**Published:** 2019-04-29

**Authors:** Christopher M Mayer, Asiphas Owaraganise, Jane Kabami, Dalsone Kwarisiima, Catherine A Koss, Edwin D Charlebois, Moses R Kamya, Maya L Petersen, Diane V Havlir, Britta L Jewell

**Affiliations:** ^1^ Albany Medical College Albany NY USA; ^2^ Infectious Diseases Research Collaboration Kampala Uganda; ^3^ Makere University Joint AIDS Program Kampala Uganda; ^4^ University of California San Francisco San Francisco CA USA; ^5^ Makere University Kampala Uganda; ^6^ University of California Berkeley Berkeley CA USA

**Keywords:** HIV, PrEP, prevention, transportation barriers, implementation science

## Abstract

**Introduction:**

Geographic and transportation barriers are associated with poorer HIV‐related health outcomes in sub‐Saharan Africa, but data on the impact of these barriers on prevention interventions are limited. We estimated the association between distance to clinic and other transportation‐related barriers on pre‐exposure prophylaxis (PrEP) uptake and initial clinic visit attendance in a rural community in southwestern Uganda enrolled in the ongoing SEARCH study (NCT01864603).

**Methods:**

Community‐wide HIV testing was conducted and offered to adult (≥15 years) participants in Ruhoko. Participants were eligible for PrEP based on an empiric risk score, having an HIV‐discordant partner, or self‐referral at either the community health campaign or during home‐based testing from March to April 2017. We collected data from PrEP‐eligible households on GPS‐measured distance to clinic, walking time to clinic and road difficulty. A sample of participants was also asked to identify their primary barriers to PrEP use with a semi‐quantitative questionnaire. We used multivariable logistic regression to evaluate the association between transportation barriers and (1) PrEP uptake among PrEP‐eligible individuals and (2) four‐week clinic visit attendance among PrEP initiators.

**Results:**

Of the 701 PrEP‐eligible participants, 272 (39%) started PrEP within four weeks; of these, 45 (17%) were retained at four weeks. Participants with a distance to clinic of ≥2 km were less likely to start PrEP (aOR 0.34; 95% CI 0.15 to 0.79, *p* = 0.012) and less likely to be retained on PrEP once initiated (aOR 0.29; 95% CI 0.10 to 0.84; *p* = 0.024). Participants who were deemed eligible during home‐based testing and did not have the option of same‐day PrEP start were also substantially less likely to initiate PrEP (aOR 0.16, 95% CI 0.07 to 0.37, *p* < 0.001). Of participants asked to name barriers to PrEP use (N = 98), the most frequently cited were “needing to take PrEP every day” (N = 18) and “low/no risk of getting HIV” (N = 18). Transportation‐related barriers, including “clinic is too far away” (N = 6) and “travel away from home” (N = 4) were also reported.

**Conclusions:**

Distance to clinic is a significant predictor of PrEP uptake and four‐week follow‐up visit attendance in a community in rural Uganda. Interventions that address geographic and transportation barriers may improve PrEP uptake and retention in sub‐Saharan Africa.

## 1 Introduction

Daily oral pre‐exposure prophylaxis (PrEP) is an efficacious HIV prevention method recommended by the World Health Organization (WHO) for persons at substantial risk of HIV infection 1. However, individuals who could benefit from PrEP may face structural barriers to PrEP uptake and retention. A number of studies have shown that geographic and transportation barriers are associated with poorer health outcomes among individuals living with HIV in sub‐Saharan Africa (SSA) 2. Distance to clinic, time to clinic and cost of transportation have all been cited as factors affecting HIV testing, antiretroviral therapy (ART) initiation, retention, adherence and mortality 3, 4, 5, 6, 7, 8, 9. For example, in a community in rural Uganda, each 10 km increase in proximity to clinic conferred a 1.5‐fold increase in the odds of retention in care 7, and greater distance to clinic has also been significantly associated with delayed or missed ART visits 6. However, data on the impact of geographic and transportation barriers for access to HIV prevention services such as PrEP are limited. Additionally, empirical measures of distance and time to clinic are largely unavailable in rural areas of SSA, where roads are often unpaved and transportation to clinic may involve crossing geographic obstacles.

Understanding key barriers to PrEP uptake and retention in SSA will be necessary for developing effective implementation strategies. In Uganda, oral PrEP is not nationally available at all public health clinics, but guidelines on the prevention and treatment of HIV and the national HIV and AIDS strategic plan indicate that PrEP scale‐up is an anticipated action towards reducing the HIV burden in the country 10, 11. Furthermore, PrEP is currently available in Uganda via ongoing demonstration and implementation projects, largely for key populations including sex workers, men who have sex with men, and high‐risk fisher folk, with plans for a future phased scale‐up 12, 13. However, structural, social and regulatory barriers to PrEP use have been identified during scale‐up in other locations in SSA, and optimal strategies for offering PrEP have not yet been clearly identified 14.

We investigated whether transportation‐related barriers were associated with PrEP uptake and four‐week PrEP clinic visit attendance among individuals offered PrEP following population‐based HIV testing in a rural Ugandan community enrolled in the ongoing SEARCH study (NCT01864603). Phase II of the study is assessing a strategy of targeted PrEP, testing and viral suppression, on top of universal test‐and‐treat, to reduce HIV incidence. This population‐based approach to PrEP initiation allows all adult, HIV‐negative individuals to be eligible for PrEP via multiple pathways, including an empirical risk score based on risk factors for prior seroconversions, having a known HIV‐positive partner, and self‐assessed risk. In this study, we hypothesized that transportation‐related factors – specifically distance to clinic, time to clinic and road difficulty – would predict both PrEP uptake and initial follow‐up visit attendance, and that participants would cite transportation factors as primary barriers to PrEP use.

## 2 Methods

### 2.1 Ethics statement

This study was approved by ethical review boards of Makere University, Uganda National Council of Science and Technology (Kampala, Uganda), Kenya Medical Research Institute (Nairobi, Kenya), and the University of California, San Francisco (USA). Participants were consented for study participation.

### 2.2 Study setting

The SEARCH study is a cluster‐randomized community trial of streamlined HIV and chronic disease testing and treatment in 32 rural communities in three regions in East Africa (eastern Uganda, southwestern Uganda and western Kenya). The intervention arm of Phase II of the study is investigating a targeted, population‐based approach to PrEP use in which individuals are eligible for PrEP via an HIV‐susceptibility risk score or by self‐assessment of risk. At baseline of Phase II in each community, SEARCH conducted hybrid mobile HIV testing, including two‐week community health campaigns (CHCs) at which participants were tested for HIV and a set of non‐communicable diseases, educated about HIV prevention and informed about PrEP, among other activities 15. Community members who did not attend the CHC were offered home‐based testing (HBT) and HIV treatment or prevention services, including PrEP, within one to six months following the CHC. We report here on PrEP uptake and week 4 follow‐up clinic visit attendance in Ruhoko, a Phase II intervention community in rural southwestern Uganda with approximately 5000 adult residents.

### 2.3 Study population

Adult (aged ≥15 years) residents of Ruhoko who were HIV‐negative were classified as eligible for PrEP if they (1) were considered at elevated risk of HIV seroconversion, based on a risk score derived by applying machine‐learning methods to HIV seroconversion data from Phase I of the trial 16 (referred to as “E”); (2) reported being in a serodiscordant relationship (referred to as “D”); or (3) self‐referred for PrEP, at either the CHC or home visits (referred to as “S”), if not identified by risk score or as being in a discordant relationship. The algorithm to develop the risk score used ensemble machine learning methods to identify demographic and behavioural characteristics – such as age, occupation, martial status, alcohol use and depression – associated with increased HIV risk 16. Sexual behaviour data were not routinely collected in SEARCH and eligibility for PrEP via the risk score was not dependent upon being formally identified as part of a key population. Risk score eligibility for PrEP was determined prior to CHC and HBT, using data from census of participants completed at the conclusion of Phase I of the trial. All risk eligibility categories in this analysis represent mutually exclusive methods of identification for PrEP eligibility, as participants deemed eligible for PrEP through the risk score or a discordant partnership were not considered to have self‐referred. In Ruhoko, the CHC was conducted from 2 to 13 March 2017, with home‐based testing occurring from 18 March to 14 April 2017.

### 2.4 PrEP intervention

Prior to the CHC, mobilization activities occurred within Ruhoko to inform participants about upcoming availability of PrEP and its HIV‐prevention benefits. SEARCH staff held meetings with village health teams, local council chairpersons, religious leaders and other local leaders, and information about PrEP was disseminated through visits to schools, churches, bars and houses. In Ruhoko, PrEP ambassadors held independent village meetings to educate community members about PrEP and how to self‐refer for PrEP use. At the CHC, eligible participants were directed to a PrEP education station where they were informed about how PrEP works, safety and efficacy, who should consider taking PrEP, and additional HIV protection while taking PrEP. If participants were interested in initiating PrEP, they were then offered referral to a linkage station to make an appointment at Ruhoko clinic for PrEP enrolment. Participants were offered the option of same‐day PrEP start or a clinic appointment at a later date. Our study included all adult Ruhoko residents assessed as eligible for PrEP at baseline of Phase II (E's, D's and S's), excluding individuals without an identifiable household location, those in prison, and those assessed as eligible after database closure on 14 June 2017. At PrEP initiation, all participants were given a one‐month supply (28 pills) of emtricitabine/tenofovir disoproxil fumarate (FTC/TDF) oral PrEP and were scheduled for a follow‐up clinic visit four weeks after initiation. All eligible participants had at least eight weeks of follow‐up after eligibility assessment to allow for complete ascertainment of initial clinic visit attendance among initiators. Participants attending a follow‐up visit were also asked to self‐report adherence to PrEP within the past three days. For participants attending the initial clinic visit, further follow‐up visits were also scheduled but were not included in this analysis.

### 2.5 Study measures

Demographic data were collected during CHC and home‐based testing for residents who did not attend CHC. Global positioning system data providing the location of each participant's household were collected during a household census enumeration conducted prior to study baseline 15. Distance (in kilometres) from participant household to clinic, time to clinic (in minutes) and road difficulty were measured between June and July 2017 by walking participant transportation routes from clinic to each PrEP‐eligible household. Distance and walking time were recorded using the Trails.io application (iosphere GmbH, Cologne, Germany) and road difficulty was assessed for each portion of the journey using the following scale: (1) flat paved or dirt roads, navigable by boda bodas (i.e. motorcycle taxis); (2) dirt roads with moderate incline, not navigable by boda bodas, some narrow and with loose gravel; (3) narrow, high‐incline dirt paths not navigable by boda bodas with large amounts of gravel and other geographic barriers (e.g. large rocks or barbed wire). Maximum road difficulty was determined as the greatest road difficulty encountered on the entire transportation route to clinic.

A sample of participants was also asked to identify participants’ primary barriers to PrEP use with a semi‐quantitative questionnaire. Part of the sample (N = 55) was stratified based on sex, risk type (E, D or S) and PrEP uptake, and the remainder (N = 49) was conducted as a convenience sample of PrEP‐eligible households.

### 2.6 Statistical analyses

We used multivariable logistic regression to evaluate the association between transportation barriers (distance to clinic, time to clinic and road difficulty) and (1) PrEP uptake among PrEP‐eligible individuals, defined as starting PrEP within four weeks of eligibility assessment; and (2) four‐week visit attendance among PrEP initiators, defined as attending a clinic visit for PrEP from two weeks before to four weeks after the expected visit date (between two and eight weeks after initiating PrEP). Distance to clinic and time to clinic were each divided into six discrete categories, with the latter five categories collapsed into one due to similar log odds and a non‐linear relationship between transportation barriers and PrEP uptake and follow‐up visit attendance. Logistic regression models for uptake were adjusted for age, sex, PrEP risk type (E, D or S), contact location (CHC or HBT) and models for four‐week clinic visit attendance were adjusted for age and eligibility criteria only, due to a smaller number of events per variable. Variables selected for inclusion in the four‐week clinic visit attendance models were chosen based on *p*‐value significance of *p* < 0.05 in the bivariate models. We also report descriptive statistics on primary barriers to PrEP use named in the questionnaire sample.

## 3 Results

### 3.1 Baseline characteristics of study participants

**Figure 1:**
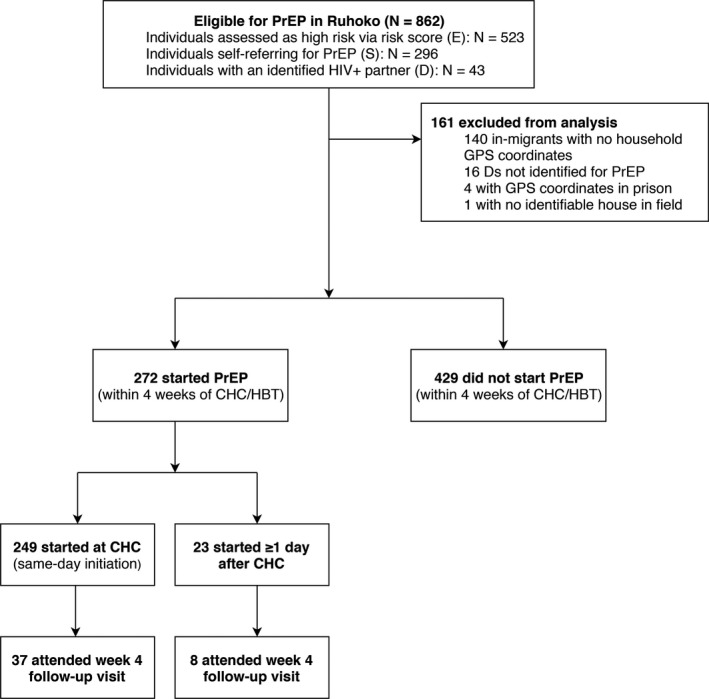
Flow chart of PrEP uptake and retention among eligible adults (aged ≥15 years). CHC, community health campaign; GPS, global positioning system; HBT, home‐based testing; PrEP, pre‐exposure prophylaxis.

**Figure 2:**
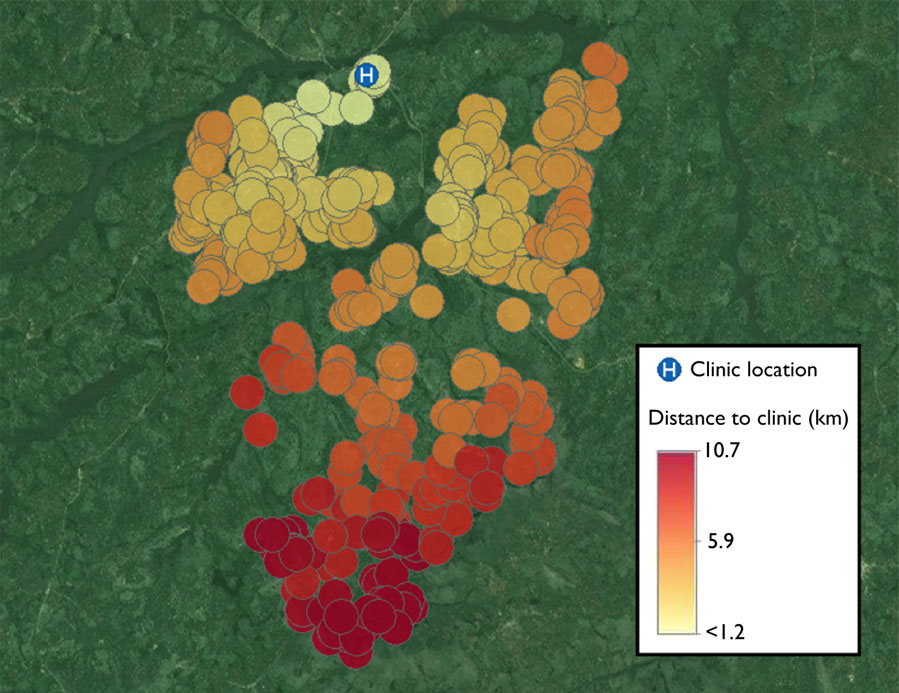
Distribution of distance to clinic for all PrEP‐eligible households in Ruhoko, Uganda based on GPS coordinates of households and walking routes (N = 701). GPS, global positioning system; PrEP, pre‐exposure prophylaxis.

As of 14 June 2017, 863 individuals were eligible for PrEP in Ruhoko, of which 701 were included in the study analysis (N = 453 (E's), N = 27 (D's) and N = 221 (S's)) (Figure 1). Median age was 26 years (IQR 22 to 31), 43% were female, and 65% were E's. PrEP‐eligible participants resided a median of 4.8 km (IQR 3.5 to 7.25) from clinic, with a median walking time of 78 minutes (IQR 56 to 111) to clinic (Figure 2). Using a 1 to 3 scale of road difficulty (1 = least difficult; 3 = most difficult), 83% of participants experienced a maximum road difficulty of 1 on their transportation route to clinic.

### 3.2 PrEP uptake and four‐week clinic visit attendance

Of the 701 individuals in the study, 39% (N = 272) started PrEP; of those who initiated PrEP, 92% (N = 249) did so on the same day as HIV testing. Uptake varied by risk group: 24% (N = 110) of E's started PrEP, compared to 63% (N = 140) of S's and 81% (N = 22) of D's. Of the PrEP initiators, 17% (N = 45) attended a clinic visit at four weeks; 8% (N = 9) of E's, 21% (N = 30) of S's and 27% (N = 6) of D's were retained. All participants who attended the four‐week follow‐up visit (N = 45) self‐reported 100% adherence to PrEP by three‐day recall.

### 3.3 Predictors of PrEP uptake and four‐week clinic visit attendance

We report unadjusted and adjusted odds of PrEP uptake among all PrEP‐eligible participants (Table 1) and four‐week clinic visit attendance among PrEP initiators (Table 2). In multivariable analysis, variables were adjusted for age, sex, PrEP eligibility group (E, D or S) and testing location (CHC or HBT). GPS distance to clinic ≥2 km was associated with both lower odds of starting PrEP and attending the first clinic visit (aOR 0.34, 95% confidence interval (CI) 0.15 to 0.79, *p* = 0.012 and aOR 0.29, 95% CI 0.10 to 0.84, *p* = 0.024 respectively). Walking time to clinic of thirty minutes or greater and higher degrees of road difficulty were also associated with decreased odds of uptake and retention, but the associations were not statistically significant. Both sex and PrEP risk type were also investigated as effect modifiers but were not significantly associated with the barriers reported.

**Table 1:** Association between parameters and PrEP uptake (among PrEP‐eligible, N = 701) in Ruhoko, Uganda

Group	N (%)	OR	*p*‐value	aOR[Link]	*p*‐value
Age					
15 to 24	339 (48%)	Reference			
25 to 34	242 (35%)	1.72 (1.21 to 2.44)	0.002	1.09 (0.74 to 1.61)	0.657
35 to 44	77 (11%)	4.49 (2.67 to 7.57)	<0.001	1.69 (0.93 to 3.07)	0.085
≥45	43 (6%)	6.64 (3.27 to 13.46)	<0.001	2.22 (1.01 to 4.86)	0.046
Sex					
Male	401 (57%)	Reference			
Female	300 (43%)	1.30 (0.95 to 1.76)	0.097	0.95 (0.67 to 1.34)	0.759
PrEP eligibility group					
E	453 (65%)	Reference			
S	221 (32%)	5.39 (3.81 to 7.63)	<0.001	4.45 (2.99 to 6.61)	<0.001
D	27 (4%)	13.72 (5.08 to 37.09)	<0.001	10.37 (3.67 to 29.36)	<0.001
Testing location					
CHC	610 (87%)	Reference			
HBT	91 (13%)	0.09 (0.04 to 0.21)	<0.001	0.16 (0.07 to 0.37)	<0.001
Distance to clinic					
<2 km	30 (4%)	Reference			
≥2 km	671 (96%)	0.35 (0.16 to 0.75)	0.007	0.34 (0.15 to 0.79)	0.012
Walking time to clinic					
<30 minutes	43 (6%)	Reference	23 (8%)	Reference	
≥30 minutes	658 (94%)	0.53 (0.28 to 0.98)	0.044	0.59 (0.30 to 1.17)	0.132
Maximum road difficulty					
1	580 (83%)	Reference	225 (83%)	Reference	
2	26 (4%)	0.84 (0.37 to 1.91)	0.669	1.17 (0.45 to 2.74)	0.810
3	95 (13%)	1.05 (0.68 to 1.64)	0.823	1.15 (0.71 to 1.87)	0.575

^1^aOR = adjusted odds ratio, adjusted for age, sex and risk type (mutually exclusive categories: E = eligible by risk score; S = eligible by self‐referral; D = eligible by having an HIV‐discordant partner). CHC = community health campaign. HBT = home‐based testing. Maximum road difficulty: 1 = low incline; navigable by boda‐bodas; 2 = moderate incline; walking only; 3 = high incline; geographic barriers; walking only.

**Table 2:** Association between parameters attendance of initial four‐week clinic visit (among PrEP initiators, N = 272) in Ruhoko, Uganda

Group	N (%)	OR	*p*‐value	aOR[Link]	*p*‐value
Age					
15 to 24	95 (35%)	Reference			
25 to 34	97 (36%)	1.89 (0.79 to 4.51)	0.153	1.29 (0.49 to 3.36)	0.605
35 to 44	49 (18%)	2.45 (0.92 to 6.51)	0.072	1.42 (0.47 to 4.30)	0.540
≥45	31 (11%)	4.55 (1.64 to 12.61)	0.004	2.46 (0.76 to 8.00)	0.134
Sex					
Male	145 (53%)	Reference			
Female	127 (47%)	1.24 (0.65 to 2.35)	0.516	0.99 (0.50 to 1.94)	0.970
PrEP eligibility group					
E	110 (40%)	Reference			
S	140 (51%)	3.06 (1.39 to 6.76)	0.006	2.37 (0.93 to 6.06)	0.070
D	22 (8%)	4.21 (1.32 to 13.42)	0.015	2.85 (0.74 to 10.89)	0.127
Testing location					
CHC	266 (98%)	Reference			
HBT	6 (2%)	1.01 (0.12 to 8.85)	0.993	1.07 (0.11 to 10.01)	0.955
Distance to clinic					
<2 km	19 (7%)	Reference			
≥2 km	253 (93%)	0.30 (0.11 to 0.82)	0.019	0.29 (0.10 to 0.84)	0.024
Walking time to clinic					
<30 minutes					
≥30 minutes	251 (92%)	0.41 (0.16 to 1.07)	0.068	0.41 (0.15 to 1.11)	0.080
Maximum road difficulty					
1					
2	9 (3%)	0.54 (0.07 to 4.47)	0.572	0.54 (0.06 to 4.60)	0.572
3	38 (14%)	0.24 (0.06 to 1.05)	0.056	0.26 (0.06 to 1.17)	0.079

^1^aOR = adjusted odds ratio, adjusted for age and risk type. CHC = community health campaign. HBT = home‐based testing. Maximum road difficulty: 1 = low incline; navigable by boda‐bodas; 2 = moderate incline; walking only; 3 = high incline; geographic barriers; walking only.

### 3.4 Barriers to PrEP use

**Figure 3:**
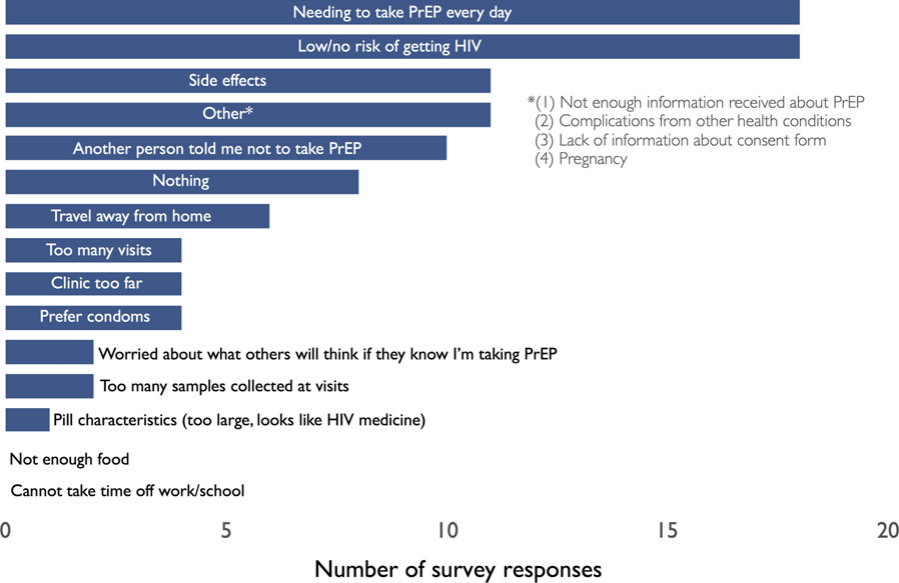
PrEP, pre‐exposure prophylaxis. Survey responses to semi‐quantitative questionnaire.

The most frequently named barriers in the questionnaire were needing to take PrEP every day (18%; N = 18) and self‐perceived low or no risk of acquiring HIV (18%; N = 18) (Figure 3). When combined, the two transportation‐related responses (“travel away from home” and “clinic too far”) were also a top barrier to PrEP use (10%; N = 10).

## 4 Discussion

In this cohort of PrEP‐eligible participants in rural southwestern Uganda, PrEP uptake was low (39%) among eligible individuals and only 17% of PrEP initiators attended the initial four‐week follow‐up clinic visit. Among the barriers to PrEP, we found that increased distance to clinic was significantly associated with decreased odds of both PrEP uptake and four‐week retention. There was a trend towards lower odds of clinic visit attendance among participants with longer walking time and increased road difficulty, but this association was not statistically significant. We also found that, when asked about primary barriers to starting or staying on PrEP, approximately 10% of participants named travel‐related reasons, although daily pill‐taking and low HIV risk perception were more frequently cited.

Our findings are consistent with previous studies on transportation‐related barriers to HIV testing and engagement in the care cascade. Both mobile and home‐based HIV testing and ART delivery have been shown to increase rates of engagement in the care cascade 17. For example, in a study in Malawi, optional home‐based initiation of ART resulted in a significant increase in the proportion of adults initiating ART in Malawi relative to facility‐based care 18. Notably, home‐based and mobile methods of testing and treatment were also able to engage individuals widely considered to be at higher risk of HIV, including men, youth and those with no prior HIV testing record 17, 19, 20. However, participants in our study reported daily pill‐taking and low risk perception as the primary barriers to PrEP use, suggesting that factors in addition to transportation may also need to be addressed to improve PrEP uptake and persistence.

Low uptake and retention of PrEP in this community is indicative of the implementation challenges that PrEP programmes in SSA may face when deployed on a population‐level, as compared to a clinical trial setting. While distance is one barrier, the overall low uptake and retention in this population show that there are many challenges facing PrEP implementation, particularly among high‐risk populations. Moreover, this also suggests that proximity to clinic is not in and of itself a strong facilitator of PrEP use for eligible individuals. Importantly, uptake and retention in our study were lower among individuals assessed to be at risk of seroconversion based on an empirically derived risk score (“E”). Both individuals self‐referring for PrEP (“S”) and those with a known HIV‐positive partner (“D”) exhibited greater uptake and retention, although there were a limited number of discordant couples in this population. An open‐label study of PrEP among mutually disclosed HIV‐serodiscordant couples in East Africa found that HIV‐negative partners exhibited high PrEP uptake, retention and adherence, with 95% initiating PrEP and 97% continuing to take PrEP after attending a first monthly visit 21. However, certain high‐risk populations (such as African‐American MSM, transgender women and young women) in other settings have demonstrated lower uptake, retention and adherence in implementation practice 22, 23, 24, 25, and low risk perception, stigma and misinformation are often cited as barriers to PrEP use 26. More work is needed to identify and address additional barriers to PrEP use, including low risk perception. In terms of implementation, this analysis contributes to other literature showing that delivery of PrEP must be optimized to improve uptake and retention, and that transportation‐related barriers must be a part of this optimization package.

A central strength of the study is that PrEP eligibility was assessed for a very high percentage of residents through a combination of CHCs and HBT, meaning that the denominator for PrEP eligibility in this community is truly population‐based. CHCs were specifically designed to be mobile within the community over a two‐week time period, and located in areas designed to facilitate transport. The majority of individuals classified as eligible for PrEP received this classification at CHC (87%), with a further 13% deemed eligible via HBT. Furthermore, immediate PrEP enrolment was offered at CHC; for many individuals, the initial transportation barrier to uptake was already removed. Thus, transportation barriers were not acting to impede uptake directly, but could have been acting indirectly through anticipated difficulty or expense of remaining in the PrEP programme in the future. Indeed, PrEP‐eligible participants reached via HBT were significantly less likely to start PrEP compared to those who had eligibility assessed at CHC (aOR 0.16, 95% CI 0.07 to 0.37, *p* < 0.001). Had same‐day start for PrEP not been provided at CHC, transportation might have been an even greater barrier to uptake.

In this study, there are also several limitations and areas for future work. We only reported time to clinic as walking time, due to lack of data on individual methods of transportation to clinic for this population. In reality, participants may have used alternative transportation methods for all or part of the journey to clinic (e.g. bicycles or boda‐bodas). In addition, our study did not report data on cost of transport to clinic, which could also be a causal factor for lower rates of uptake and retention of PrEP, particularly given that travel vouchers were not provided for PrEP clinic visits. Due to low retention at four weeks, we also have limited power to determine the effect of transportation barriers and other covariates on retention. Furthermore, as this analysis focused on initial clinic visit attendance, the role of distance as a barrier may be different for long‐term retention among those individuals who started PrEP and returned at four weeks. While other timepoints were assessed, low numbers of continuing PrEP users at four weeks also limited our ability to assess the role of distance beyond the initial visit. Finally, the analysis was limited to a single community in southwestern Uganda, and findings may not be applicable to other communities or settings. For example, in Phase I of SEARCH, participants travelling to clinic for initial linkage to care for ART or non‐communicable disease care were provided a travel voucher equivalent to the approximate cost of one‐way travel to clinic 15, 27. Participants in SEARCH communities may have grown accustomed to services provided by the trial, and therefore could have different barriers to accessing PrEP than other individuals using PrEP outside of the context of a clinical trial. The role of distance as a potential barrier to PrEP use may also be highly population‐specific, and could vary considerably in other settings with different geographic landscapes, local transportation options or the strength of other barriers.

## 5 Conclusions

In order to design effective implementation strategies for PrEP in sub‐Saharan Africa, it is necessary to understand barriers to uptake, retention and adherence. Our study shows that among eligible individuals PrEP uptake and four‐week clinic visit attendance were low and that transportation‐related factors may be an important obstacle to initial PrEP use. Further work should be done to investigate ways to address these and other barriers to improve PrEP rollout.

## Competing interests

The authors declare no conflicts of interest.

## Authors’ contributions

CMM, MLP, DVH and BLJ designed the study. CMM collected data on distance to clinic, time to clinic and road difficulty. CMM and BLJ analysed and interpreted the data with input from MLP and DVH. CMM and BLJ wrote the manuscript with input from all authors, including AO, JK, DK, CAK, EDC, MRK, MLP and DVH.
